# Untargeted Lipidomics Reveals Lipid Alterations in Colonic Contents of C57BL/6J Mice with Acute *Toxoplasma gondii* Infection

**DOI:** 10.3390/microorganisms14092077

**Published:** 2026-09-17

**Authors:** Wen-Jie Cheng, Bo-Fang Duan, Cai-Qin Deng, Peng-Hao Wei, Min-Min Sui, Yi-Dan Wang, Xing-Quan Zhu, Feng-Cai Zou, Zhao Li

**Affiliations:** 1 Faculty of Animal Science and Technology, Yunnan Agricultural University, Kunming 650201, China; cwj210365@163.com (W.-J.C.); weipenghao0414@163.com (P.-H.W.); smm9797@163.com (M.-M.S.); wangyidan318@163.com (Y.-D.W.); 2 First People’s Hospital of Kunming City, Kunming 650032, China; 3 The Yunnan Key Laboratory of Veterinary Etiological Biology, College of Veterinary Medicine, Yunnan Agricultural University, Kunming 650201, China; askalm@163.com (B.-F.D.); dengcaiqin1998@163.com (C.-Q.D.); xingquanzhu1@hotmail.com (X.-Q.Z.); 4 Yunnan Center for Animal Disease Prevention and Control, Kunming 650201, China; 5 Animal Research and Resource Center, School of Life Sciences, Yunnan University, Kunming 650500, China; 6 Shanxi Key Laboratory of Animal Disease Research, College of Veterinary Medicine, Shanxi Agricultural University, Jinzhong 030801, China

**Keywords:** *Toxoplasma gondii*, acute infection, lipidomics, colonic contents

## Abstract

Background: Although *Toxoplasma gondii* has been recognized as an obligate intracellular parasite, the lipid metabolic alterations it induces in colonic contents during acute infection remain poorly characterized. Methods: An acute infection model was established using C57BL/6J mice by oral inoculation with the *T. gondii* ME49 strain. Untargeted lipidomics analysis was performed on the colonic contents collected at day 10 post-infection. Results: Acute infection significantly elevated glycerophospholipids (GPs) and reduced glycerolipids (GLs), and prenol lipids (PRs), leading to 136 differentially abundant lipids. Pathway enrichment analysis identified the two most significantly affected pathways: choline metabolism in cancer and glycerophospholipid metabolism, with lysophosphatidic acid (LPA(16:0)) and lysophosphatidylcholine (LPC(18:1)) functioning as core hub nodes. Receiver operating characteristic (ROC) analysis identified phosphatidylethanolamine (PE(20:1e_22:4)), PE(16:0_22:6), hexosylceramide (Hex1Cer(m18:0_20:4)), and Hex1Cer(t17:0_22:6) as exploratory candidate biomarkers with high discriminatory performance (AUC > 0.94). Conclusions: Acute *T. gondii* infection induces lipid metabolic reprogramming in colonic contents of C57BL/6J mice. The findings provide a novel metabolic perspective on the intestinal pathogenesis of toxoplasmosis and offer exploratory candidate lipid markers for diagnosing acute infection.

## 1. Introduction

As an obligate intracellular parasitic protozoan of the phylum Apicomplexa, *Toxoplasma gondii* infects nearly all warm-blooded animals, including humans [1]. It is widely distributed worldwide and is considered one of the most adaptable parasites [2]. *T. gondii* infects humans primarily through the ingestion of oocyst-contaminated water or food, the consumption of undercooked or raw meat with tissue cysts, or congenital vertical transmission [3,4]. Following *T. gondii* infection, immunocompetent individuals usually remain asymptomatic or show subclinical infection, whereas lymphadenopathy, pneumonia, and severe neurological disorders may manifest in immunocompromised individuals [5,6]. Fetal infection via the placenta can lead to congenital toxoplasmosis with severe outcomes such as developmental abnormalities and even miscarriage [4,7]. Thus, elucidating the interactions between *T. gondii* and its hosts is of great significance for clarifying its pathogenic mechanisms.

The intestine is the primary site of *T. gondii* infection following oral intake [8], where it produces bradyzoites or sporozoites that invade the intestinal mucosa, which rapidly convert into tachyzoites that proliferate extensively within intestinal epithelial cells [9]. *T. gondii* proliferation triggers local inflammatory responses that impair intestinal barrier function and alter the expression of tight junction proteins [10]. Intestinal inflammation is often not confined to the small intestine but can spread downward to the colon, where it significantly alters the colonic microenvironment and causes mucosal barrier damage, immune cell infiltration, and abnormal accumulation of microbial metabolites [11,12]. *T. gondii* infection also significantly remodels gut microbiota composition and function, thereby affecting host metabolic homeostasis and immune response [13,14]. Rich in unabsorbed metabolites, shed epithelial cells, and secondary metabolites from commensal microorganisms, colonic contents can reflect the interactions between the host and the gut microecosystem [15]. Deciphering the metabolic reprogramming in colonic contents induced by *T. gondii* infection can help reveal the molecular-level intestinal pathological changes and host–parasite interaction mechanisms.

Lipidomics, a branch of metabolomics, focuses on identifying lipid species, their structures, and their dynamic changes [16]. While serving as fundamental cell membrane components, lipids are also involved in critical biological processes such as energy storage, signal transduction, and immune responses [17]. High-throughput lipidomics based on liquid chromatography-tandem mass spectrometry (LC-MS/MS) has seen wide applications in disease mechanism exploration [18]. Previous metabolomics research showed that the lipid profiles and amino acid pathways in host serum undergo significant alterations following *T. gondii* infection [19]. Nevertheless, systematic research on intestinal contents, particularly the colonic luminal lipidome, remains relatively scarce. Comprehensive analysis of lipidomic changes in colonic contents during *T. gondii* infection can provide valuable insights into infection-induced luminal metabolic disturbances and potential lipid signaling pathway abnormalities [20].

In this study, C57BL/6J mice were orally infected with *T. gondii*, and colonic contents were collected during acute infection for untargeted lipidomics analysis. The lipid species and metabolic pathways of the acute infection group and the control were compared. The host–parasite interaction mechanisms at the colonic lipid metabolic interface were elucidated to provide new experimental evidence for clarifying the intestinal pathogenesis of toxoplasmosis and related lipid interactions.

## 2. Materials and Methods

### 2.1. Ethics Statement

This study received formal approval by the Institutional Animal Care and Use Committee of Yunnan University (Approval No. 20241024). All procedures were conducted in accordance with the national guidelines for the care and use of laboratory animals in China.

### 2.2. Chemicals and Reagents

Liquid chromatography-mass spectrometry (LC-MS) grade methanol (A454-4) and acetonitrile (A998-4) were purchased from Thermo Fisher Scientific (Waltham, MA, USA). Honeywell Fluka (Morris Plains, NJ, USA) supplied the ammonium formate (17843-250G), and DIMKA (Philadelphia, PA, USA) supplied the formic acid (50144-50 mL).

### 2.3. Animal Experiments

Specific pathogen-free (SPF) female ICR and female C57BL/6J mice (6 to 8 weeks old) were supplied by the Animal Research and Resource Center of Yunnan University (CNAS LA0029). The animal use protocol and ethics were reviewed and approved by the Institutional Animal Care and Use Committee of Yunnan University (Approval No. YNU20241024; date: 2 February 2024). Prior to the experiments, the animals were acclimatized for one week under standard SPF conditions. Mice were kept under controlled environmental conditions at 22 ± 2 °C, 50% to 60% relative humidity, and a 12 h light/dark photoperiod, with free access to food and water. The animal rooms were maintained in accordance with the Chinese national standard GB 14925-2023 Laboratory Animal—Requirements of Environment and Housing Facilities. Tachyzoites of the *T. gondii* ME49 strain (type II), initially kept in liquid nitrogen, were thawed and utilized for human foreskin fibroblasts (HFFs) infection. At 48 to 60 h post-infection, tachyzoites were collected, purified, and counted [21]. Then, 500 freshly collected tachyzoites were intraperitoneally injected into each ICR mouse [22]. The ICR mice were used exclusively for parasite propagation to obtain sufficient brain cysts and were not used for lipidomics analysis. At 30 days post-infection, ICR mice were euthanized. Brain tissues were then collected and homogenized. The C57BL/6J mice were used for the infection experiment and subsequent lipidomics analysis. Female C57BL/6J mice were infected using tissue cysts from ICR mouse brains. A total of 12 C57BL/6J mice were randomly allocated to two groups: an infected group (CJA, brain cyst administration, *n* = 6) and a control group (CJN, brain cyst negative, *n* = 6). Through oral gavage, 100 μL of PBS suspension containing 100 brain cysts was administered to the infected group, whereas each control group subject received the same volume of PBS alone. Our previous work systematically characterized this infection model, assessing parasite burden by qPCR (CT values) at multiple time points in orally infected C57BL/6J mice [22]. Inclusion criteria were healthy female C57BL/6J mice aged 6–8 weeks that completed one week of acclimatization; no exclusion criteria were applied, and all enrolled animals were included in the final analysis. All 12 C57BL/6J mice (6 per group) survived until the end of the experiment; no mortality was observed, and thus no humane endpoints were triggered. All animals originally enrolled were included in the final analysis. The experiment was not blinded, as the same investigator performed both the gavage and sample processing. However, all samples were coded before lipidomics analysis, so the operator was unaware of group allocation during data acquisition.

### 2.4. Colonic Content Collection and Storage

On day 10 post-infection (acute infection phase), CO_2_ asphyxiation was used to euthanize the C57BL/6J mice. Colonic contents were immediately collected and frozen in liquid nitrogen before storing at −80 °C for further lipidomics analysis.

### 2.5. Metabolite Extraction

Briefly, 25 mg of colonic contents was loaded in a 2 mL polypropylene tube, and two small magnetic beads were added. A mixture of internal standards, including 15:0-18:1(d7) phosphatidylcholine (PC), 18:1-d7 lysophosphatidylethanolamine (Lyso PE), and 15:0-18:1(d7) phosphatidylserine (PS), was spiked into each sample prior to extraction for quality control and normalization purposes. Thereafter, 800 µL of pre-chilled dichloromethane/methanol (3:1, *v*/*v*) was supplemented. Following a 5 min grind, the homogenate was ultrasonicated on ice for 10 min and stored at −20 °C. Following centrifugation at 25,000× *g* for a duration of 15 min at 4 °C, the supernatant (600 µL) was evaporated to dryness by lyophilization. The sample was resuspended with 600 µL of a prepared solvent system (isopropanol:acetonitrile:water = 2:1:1, *v*/*v*/*v*), vortexed for 10 min, and ultrasonicated under ice-cold conditions for 10 min. The derived homogenate was subjected to centrifugation at 25,000× *g* for 15 min at 4 °C, and the resulting supernatant was employed for UPLC-MS analysis. For quality control, 20 µL from each test sample was pooled.

### 2.6. UPLC-MS Analysis

Lipidomic analysis was facilitated by a CSH C18 column (1.7 μm, 2.1 × 100 mm, Waters, Milford, MA, USA) and a Q Exactive mass spectrometer (Thermo Fisher Scientific, Waltham, MA, USA). QC samples were injected every four experimental samples throughout the analytical run to monitor system performance. In positive ion mode, solvent A in the mobile phase consisted of acetonitrile/water (60:40, *v*/*v*) with 10 mmol/L ammonium formate and 0.1% formic acid. Solvent B in the mobile phase comprised acetonitrile and isopropanol (10:90 *v*/*v*) supplemented with ammonium formate (10 mmol/L) and formic acid (0.1%). In negative ion mode, solvent A in the mobile phase contained acetonitrile/water (60:40, *v*/*v*) supplemented with ammonium formate (10 mmol/L). Solvent B in the mobile phase was made up of isopropanol/acetonitrile (90:10, *v*/*v*) supplemented with ammonium formate (10 mmol/L). The mobile phase gradient program was as described below: 0–2 min, 40% to 43% B; 2–2.1 min, 43% to 50% B; 2.1–7 min, 50% to 54% B; 7–7.1 min, 54% to 70% B; 7.1–13 min, 70% to 99% B; 13–13.1 min, 99% to 40% B; and 13.1–15 min, 40% B. A flow rate of 0.4 mL/min, a column temperature of 55 °C, and an injection volume of 5 μL were used. Data-dependent acquisition (DDA) was employed on the Q Exactive mass spectrometer to record MS1 and MS/MS data.

Data processing and relative quantification were conducted with LipidSearch v.4.1 (Thermo Fisher Scientific, Waltham, MA, USA), with lipid annotation confidence level 2 (putatively annotated based on MS/MS spectral matching) according to the Metabolomics Standards Initiative guidelines. Mass accuracy tolerance, MS/MS acquisition and spectral matching procedures, and lipid annotation steps were adopted from previous studies [23]. Data preprocessing, including peak alignment (retention time deviation ≤ 0.1 min) and baseline correction, was performed using metaX v.1.4.0, and lipid features with ≥50% missing values across samples were excluded. Missing values were imputed using half of the minimum positive value per lipid feature. QC sample evaluation criteria included retention time stability (deviation ≤ 0.1 min), signal variation (coefficient of variation, CV ≤ 30% for ≥80% of lipid features), and PCA clustering performance. For normalization, data were log2-transformed and normalized by total ion current (TIC), followed by Pareto scaling for principal component analysis (PCA), partial least squares discriminant analysis (PLS-DA), and orthogonal PLS-DA (OPLS-DA). The models were established using seven-fold cross-validation to assess predictive performance. Model robustness was evaluated via 200 permutation tests, with R^2^ and Q^2^ values reported as indicators of goodness-of-fit and predictive ability.

### 2.7. Statistical Analysis

All data are expressed as mean ± standard deviation (SD). Differences in group means were analyzed using SPSS v 20.0 (IBM, Armonk, NY, USA). Figures were plotted in GraphPad Prism 8. False discovery rate (FDR) correction was applied using the Benjamini–Hochberg method to control for multiple comparisons. Differentially abundant lipids were selected based on variable importance in projection (VIP) > 1, fold change (FC) ranging from 0.83 to 1.2, and an FDR-adjusted *q* < 0.05.

## 3. Results

### 3.1. Quality Control

The QC samples exhibited highly consistent retention times and peak areas in both positive and negative ion modes when overlaying their total ion chromatograms. Hence, the analytical system can guarantee satisfactory repeatability and stability for subsequent experiments (Figure S1A,B). In terms of quantitative repeatability, the proportion of metabolites with CV < 30% was >0.6, indicating acceptable overall data quality (Figure S1C).

### 3.2. Lipid Composition of Colonic Contents

**Figure 1:**
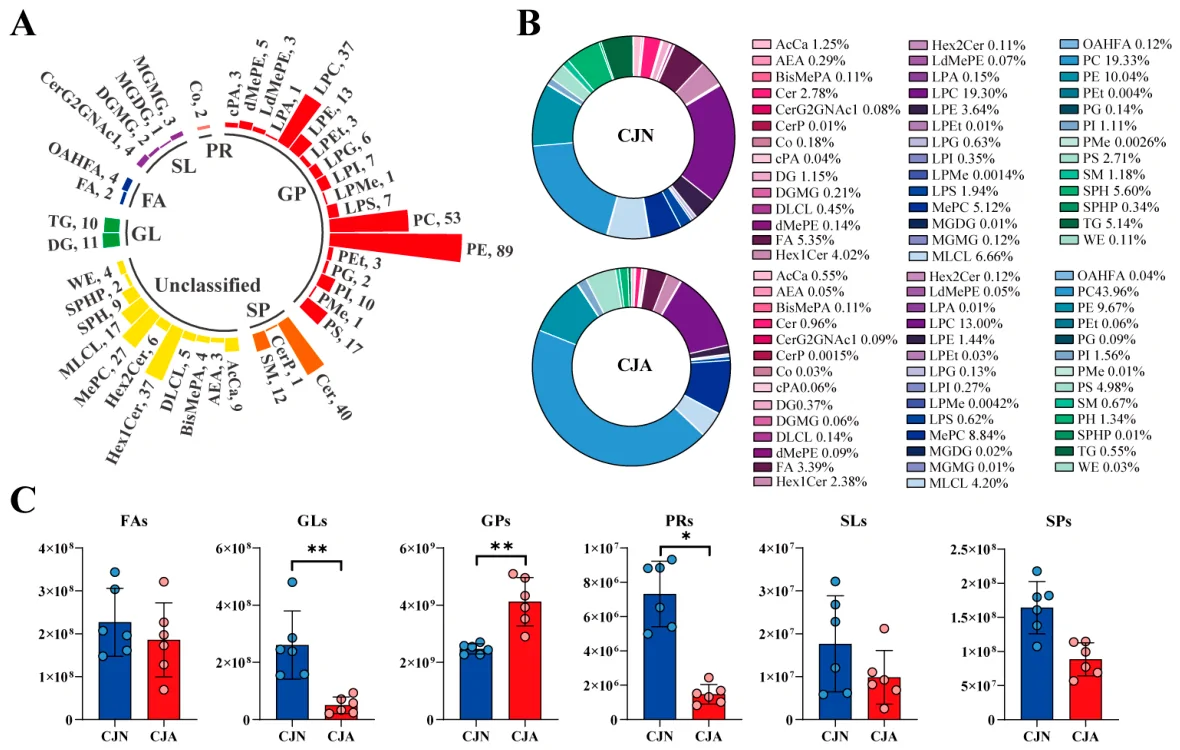
Colonic lipid composition in CJA and CJN groups (*n* = 6 per group). (**A**) Classification of lipid species identified in colonic contents. Different lipid classes are represented by distinct colors in the bar graph: glycerophospholipids (GPs), sphingolipids (SPs), glycerolipids (GLs), saccharolipids (SLs), fatty acyls (FAs), and prenol lipids (PRs). The abbreviations shown in parentheses correspond to specific lipid subclasses, and the accompanying numbers indicate the number of differentially abundant lipid species in each subclass. (**B**) Percentage composition of lipid subclasses in CJA and CJN groups. (**C**) Comparison of major lipid categories between CJA and CJN groups. Data are presented as mean ± SD. Statistical significance was determined by Student’s *t*-test with FDR correction (*q*-values). * *q* < 0.05, ** *q* < 0.01. CJA: acute *T. gondii* infection group; CJN: uninfected control group.

UPLC-MS-based lipidomic analysis was performed on colonic content samples from the CJA and CJN groups. A total of 476 lipid species in 41 subclasses were identified (Supplementary Table S1). Phosphatidylethanolamine (PE), phosphatidylcholine (PC), and ceramide (Cer) emerged as the main subclasses, encompassing 89, 53, and 40 of the identified lipids, respectively. Glycerophospholipids (GPs; 261) showed the greatest abundance, followed by sphingolipids (SPs), glycerolipids (GLs), saccharolipids (SLs), fatty acyls (FAs), and prenol lipids (PRs), encompassing 53, 21, 10, 6, and 2 lipids, respectively (Figure 1A). The CJA and CJN groups shared the same lipid subclasses, whereas the relative proportion of individual subclasses varied markedly (Figure 1B). Relative to the CJN group, the CJA group exhibited lower relative abundances of lysophosphatidylcholine (LPC) (19.30% vs. 13.00%), monolysocardiolipin (MLCL) (6.66% vs. 4.20%), sphingosine (SPH) (5.60% vs. 1.34%), triglyceride (TG) (5.14% vs. 0.55%), and fatty acid (FA) (5.35% vs. 3.39%) but increased levels of methylated phosphatidylcholine (MePC) (5.12% vs. 8.84%), phosphatidylcholine (PC) (19.33% vs. 43.96%), and phosphatidylserine (PS) (2.71% vs. 4.98%). Following *T. gondii* infection, the abundance of GP increased significantly in the CJA group (*q* < 0.01), whereas the GL, and PR levels decreased significantly (*q* < 0.05; Figure 1C).

### 3.3. Multivariate Data Analysis of Lipid Profiles in Colonic Contents

**Figure 2:**
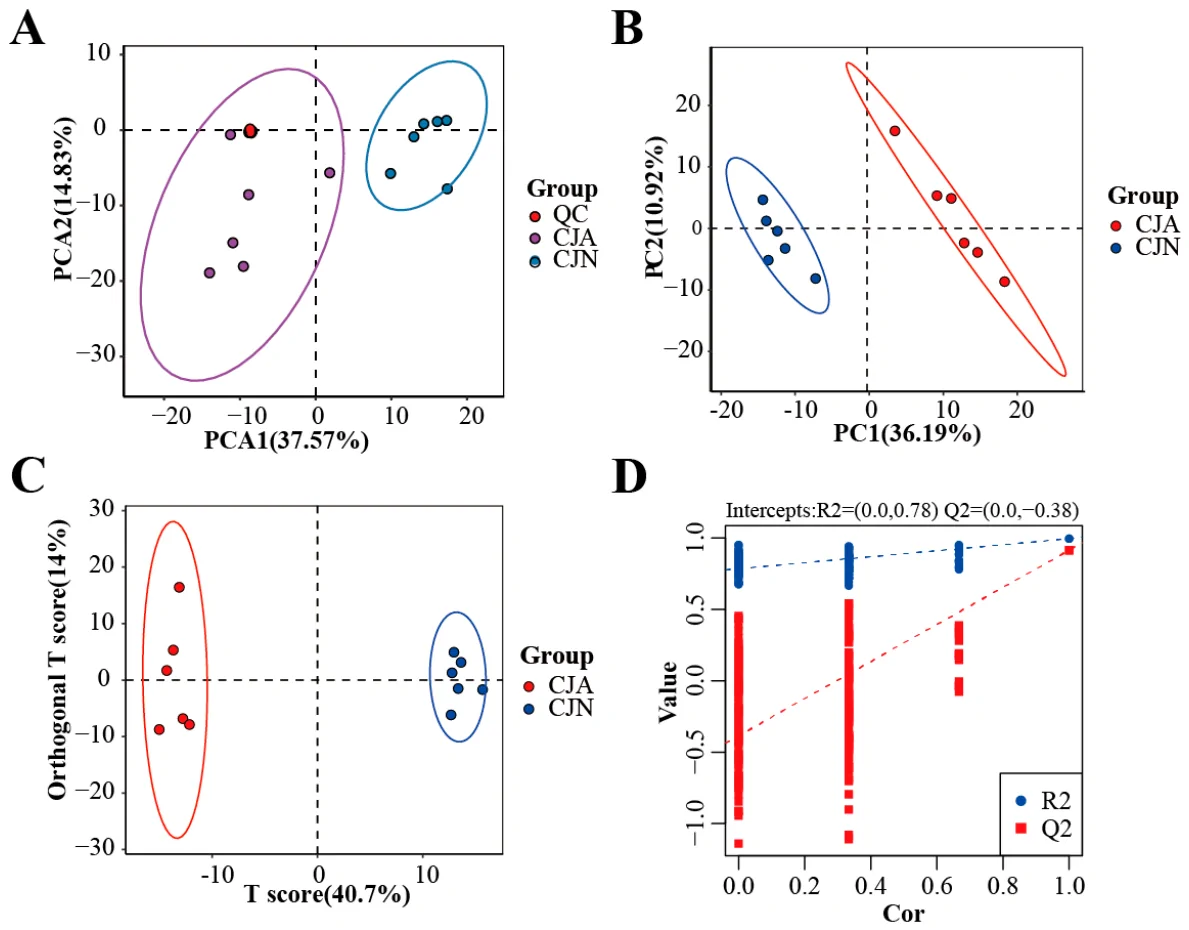
Data analysis of colonic lipid profiles in CJA and CJN groups (*n* = 6 per group). (**A**) PCA score plot comparing the between CJA and CJN groups. (**B**) PLS-DA score plot comparing samples from the CJA and CJN groups. (**C**) OPLS-DA score plot comparing the two groups. (**D**) Permutation test (200 permutations) for OPLS-DA model validation, with R^2^Y = 0.995 and Q^2^ = 0.914, indicating no overfitting and good predictive ability. PCA, PLS-DA, and OPLS-DA models were established using seven-fold cross-validation with Pareto scaling. QC: quality control; CJA: acute *T. gondii* infection group; CJN: uninfected control group.

PCA clearly discriminated the CJA group from the CJN group. Meanwhile, the close clustering of the QC samples confirmed the analytical system as stable and reliable (Figure 2A). PLS-DA revealed distinct clustering of the CJA and CJN groups, reflecting the significant impact of *T. gondii* infection on the colonic lipid profile (Figure 2B). OPLS-DA was performed for supervised pattern recognition to discriminate between CJN and CJA. The two groups showed complete separation in the OPLS-DA score plot (Figure 2C). Model reliability was evaluated via permutation tests, yielding R^2^Y = 0.995 and Q^2^ = 0.914. The cumulative R^2^Y and Q^2^ values derived from the permutation test indicated that the model was not overfitted, with Q^2^ exceeding the 0.9 threshold, confirming good predictive ability and robustness (Figure 2D). These results demonstrate that acute *T. gondii* infection markedly remodeled the colonic lipid metabolic landscape in mice.

### 3.4. Identification of Lipid Biomarkers

**Figure 3:**
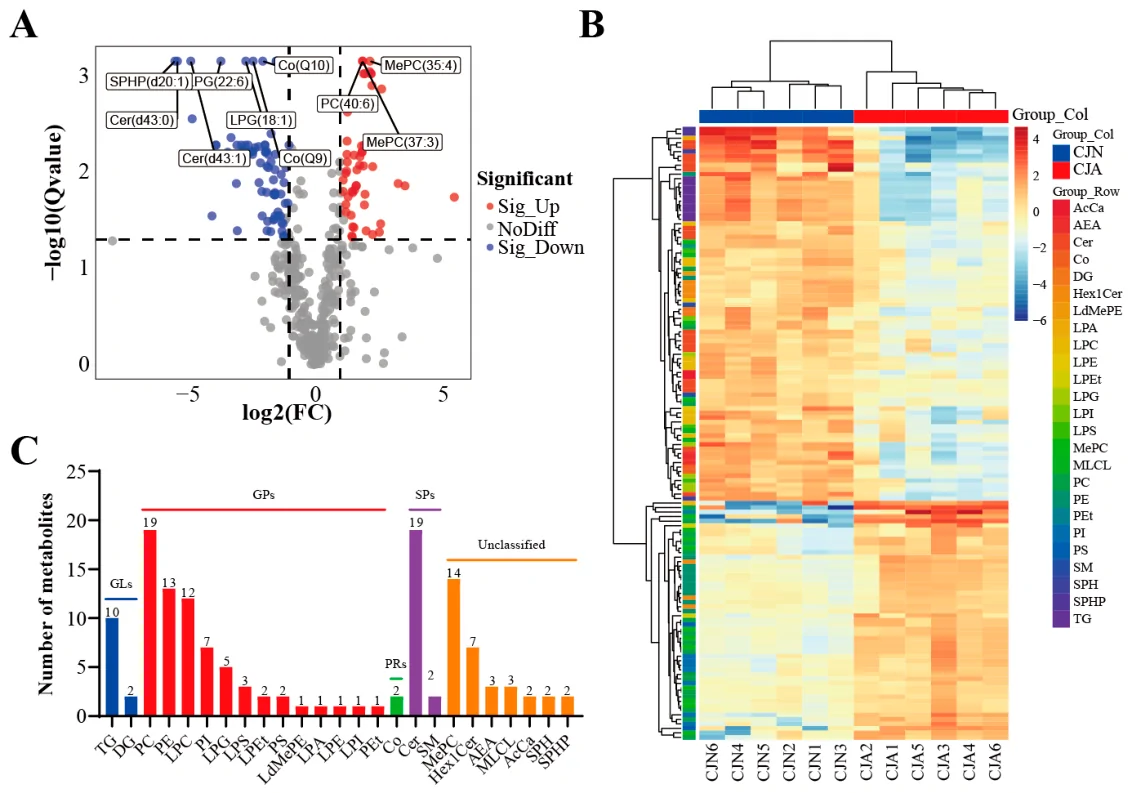
Identification of differentially abundant lipid species in colonic contents of mice following acute *T. gondii* infection (*n* = 6 per group). (**A**) Volcano plot of differentially abundant lipids between CJA and CJN groups. Red and blue dots represent up-regulated (53 species) and down-regulated (83 species) lipids, respectively, based on VIP > 1, FC > 1.2 or < 0.83, and FDR-adjusted *q* < 0.05. The x-axis represents log_2_(fold change), and the y-axis represents −log_10_(FDR-adjusted *q*-value). The horizontal dashed line indicates the significance threshold of FDR-adjusted *q* = 0.05, and the vertical dashed lines indicate the fold-change thresholds of log_2_(FC) = log_2_(1.2). (**B**) Heatmap of colonic differentially abundant lipids between CJA and CJN groups. Each column represents an individual sample. Each column represents an individual sample. (**C**) Bar graph of lipid subclasses among the 136 differentially abundant lipids.

Differentially abundant lipids associated with *T. gondii* infection were screened to identify potential lipid biomarkers according to the thresholds of VIP > 1, FC ranging from 0.83 to 1.2, and *q* < 0.05. The screening yielded 136 lipids as candidate biomarkers associated with *T. gondii* infection. Compared with the CJN group, the CJA group exhibited 53 upregulated and 83 downregulated lipid species (Figure 3A; Supplementary Table S2). The heatmap further illustrated the changes in lipids within colonic contents of C57BL/6J mice following acute *T. gondii* infection (Figure 3B). These lipids included 19 Cer, 19 PC, 14 MePC, 13 PE, 12 LPC, and 10 TG species (Figure 3C).

### 3.5. Enrichment Analysis of Differentially Abundant Lipid Metabolic Pathways in Colonic Contents

**Figure 4:**
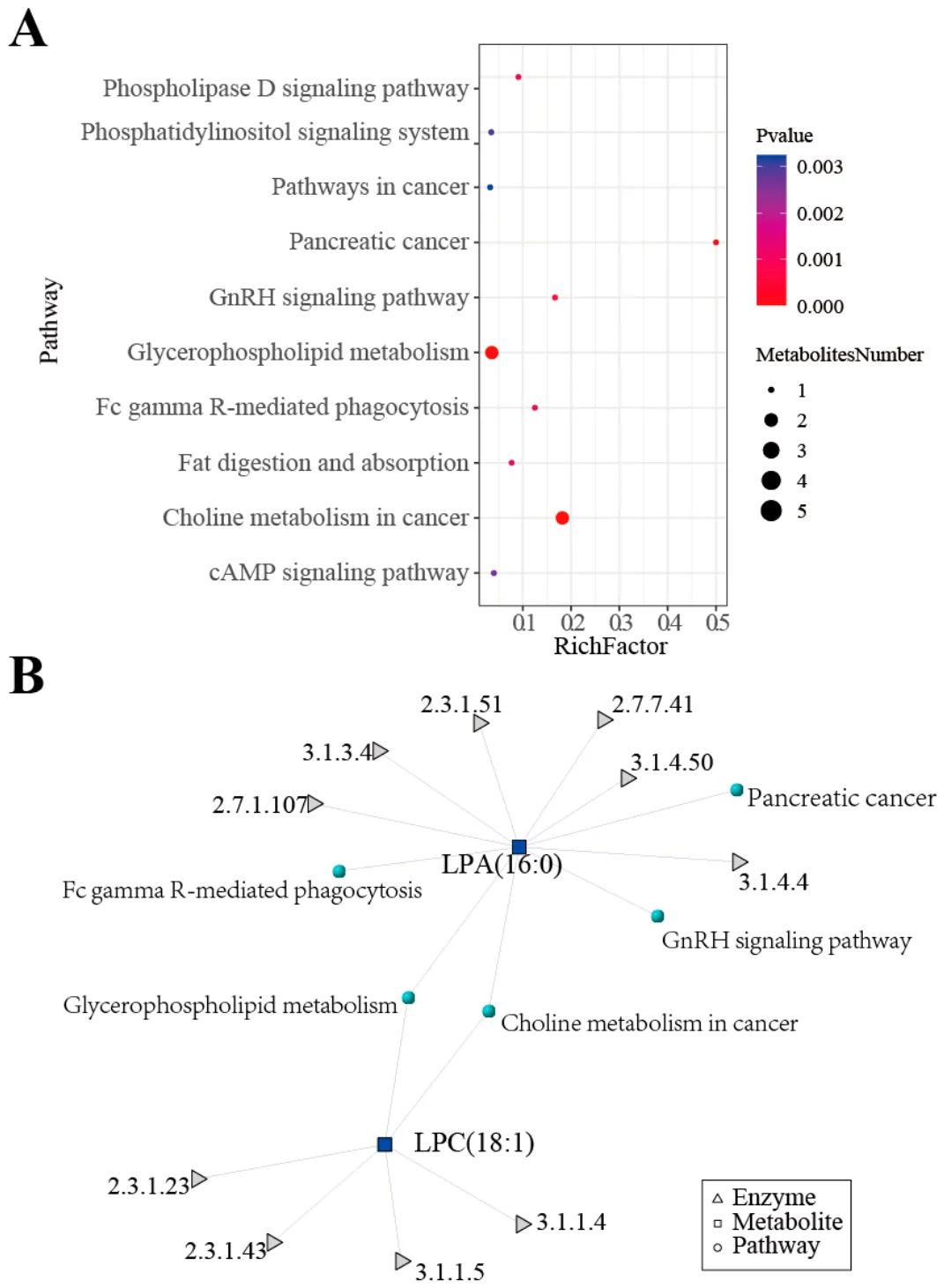
Enrichment analysis of metabolic pathways associated with the differentially abundant lipids (**A**) and network analysis (**B**). Pathway enrichment was performed using the KEGG database, and *q* < 0.05 (FDR-corrected) was considered statistically significant. (**B**) Network analysis integrating differential lipids, pathways, and enzymes. Node colors indicate different entity types (lipids, pathways, or enzymes).

Pathway enrichment analysis was conducted using the KEGG database. Various lipids were significantly enriched in the following pathways: Choline metabolism in cancer, GP metabolism, Fc gamma R-mediated phagocytosis, GnRH signaling pathway, and Pancreatic cancer. The significantly enriched lipids included LPA(16:0) and LPC(18:1) (Figure 4A). Network analysis revealed LPA(16:0) and LPC(18:1) as core nodes connecting multiple metabolic pathways (Figure 4B). The associated enzymes in the network included phospholipase D (3.1.4.4), phospholipase A2 (3.1.1.4), lysophospholipase (3.1.1.5), phosphatidate phosphatase (3.1.3.4), and diacylglycerol kinase (2.7.1.107). These findings indicate that *T. gondii* infection significantly disrupted GP metabolism and membrane lipid homeostasis in the colon.

### 3.6. Identification of Differentially Abundant Lipids in Colonic Contents of Mice with Acute Toxoplasmosis

**Figure 5:**
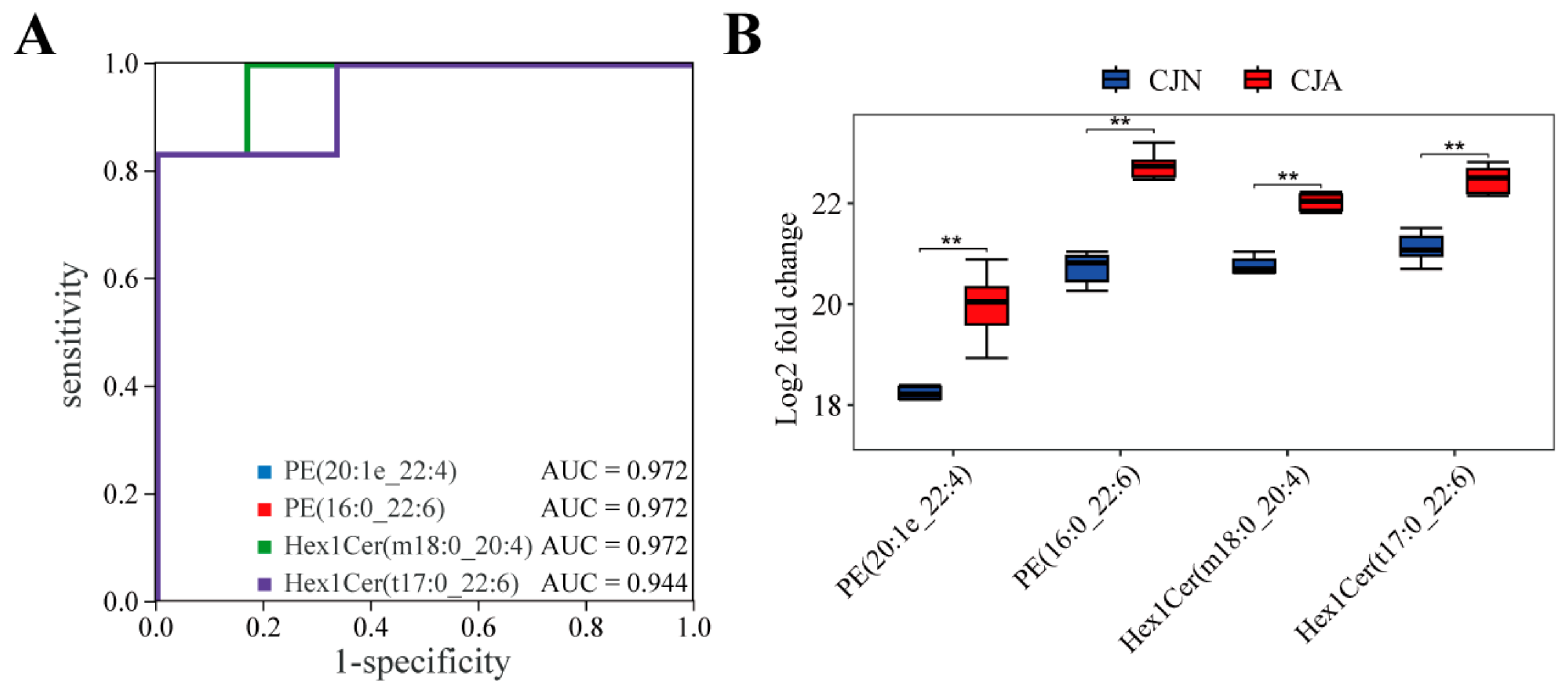
Identification of differentially abundant lipids as exploratory candidate biomarkers for acute toxoplasmosis (*n* = 6 per group). (**A**) ROC curves of four candidate lipids: PE(20:1e_22:4), PE(16:0_22:6), Hex1Cer(m18:0_20:4), and Hex1Cer(t17:0_22:6). The AUC values for PE(20:1e_22:4), PE(16:0_22:6), and Hex1Cer(m18:0_20:4) were all 0.9722. Because these three ROC curves completely overlapped, only the topmost green curve (Hex1Cer(m18:0_20:4)) is visible, while the blue (PE(20:1e_22:4)) and red (PE(16:0_22:6)) curves are obscured beneath it. Hex1Cer(t17:0_22:6) achieved an AUC of 0.9444. (**B**) Relative abundances of the four candidate lipids in colonic contents of the CJA and CJN groups. Data are presented as mean ± SD. Statistical significance was determined by Student’s *t*-test with FDR correction. ** *q* < 0.01. CJA: acute *T. gondii* infection group; CJN: uninfected control group.

The discriminatory performance of the candidate lipids in distinguishing between *T. gondii*-infected and control mice was further evaluated using receiver operating characteristic (ROC) analysis. Four lipids (PE(20:1e_22:4), PE(16:0_22:6), Hex1Cer(m18:0_20:4), and Hex1Cer(t17:0_22:6)) exhibited good predictive capacity for colonic *T. gondii* infection in mice. The area under the curve (AUC) for PE(20:1e_22:4) (95% CI: 0.8889–1.000, sensitivity: 1.000, specificity: 0.833), PE(16:0_22:6) (95% CI: 0.8597–1.000, sensitivity: 1.000, specificity: 0.833), and Hex1Cer(m18:0_20:4) (95% CI: 0.8333–1.000, sensitivity: 1.000, specificity: 0.833) was all 0.9722, while Hex1Cer(t17:0_22:6) achieved an AUC of 0.9444 (95% CI: 0.7500–1.000, sensitivity: 0.833, specificity: 1.000) (Figure 5A). Thus, all four lipids were significantly upregulated in the colon of infected mice (*q* < 0.01, Figure 5B).

## 4. Discussion

Leveraging untargeted lipidomics, this study comprehensively characterized alterations in the lipid profile of colonic contents of C57BL/6J mice following acute *T. gondii* infection, thereby depicting the luminal lipid landscape during infection. The results demonstrated that acute *T. gondii* infection significantly remodeled the lipid composition of colonic contents, particularly GPs and GLs, which is consistent with previous observations in BALB/cJ mice [20]. These experimental findings further clarified the host–parasite interactions from the perspective of intestinal lipid metabolism during *T. gondii* infection.

GPs of the CJA group exhibited a significant elevation in abundance following infection, whereas GLs, and PRs were significantly decreased. Thus, *T. gondii* infection selectively affects lipid metabolism [24]. This lipid alteration pattern is consistent with previous findings in BALB/cJ mice [20]. Therefore, the class-selective impact of *T. gondii* infection on colonic lipid metabolism is robust across different mouse strains and independent of host genetic background. *T. gondii* infects by relying on its precise manipulation of host metabolism. Lipid metabolism is particularly important among the involved metabolic pathways, given its direct association with membrane structural remodeling, signal transduction cascades, and immune response regulation [25]. GPs are primary components of cellular membrane phospholipids [26]. *T. gondii* infection was demonstrated to impair intestinal barrier function, downregulate tight junction protein expression, and damage the mucosal layer [10,27]. These alterations could potentially trigger substantial membrane phospholipid release into the colonic contents, which represents one possible mechanism for the elevated GP levels observed in the infected group. This interpretation is consistent with the hypothesis that luminal GP elevation might partly originate from shed epithelial membranes. However, this remains a speculative mechanism that would require tissue-specific evidence for direct confirmation. GPs also serve as precursors for multiple inflammatory signaling pathways, and their metabolites (e.g., lysophosphatidic acid (LPA) and arachidonic acid) are involved in regulating inflammatory responses and immune cell recruitment [28,29]. Therefore, elevated GPs in colonic contents may simultaneously reflect infection-induced intestinal structural damage and the intensity of local inflammatory responses [30]. SP metabolism was also examined, and SP levels in the infected group showed a trend toward decrease, although this difference did not reach statistical significance after FDR correction. This represents a direct observation of a non-significant trend at the lipid class level. As important components of eukaryotic cell membranes, SPs are widely involved in various signal transduction pathways [31]. Their decline may be associated with *T. gondii*’s ability to acquire SPs by hijacking host Golgi-derived vesicles to support its proliferation [32]. This hypothesis, while biologically plausible, requires further experimental validation. Thus, decreased SPs in colonic contents may reflect depleted host SP levels following extensive parasite uptake, suggesting *T. gondii* interference with host SP homeostasis driven by its own nutritional needs.

CJA and CJN shared identical lipid subclass compositions, but the relative abundances of individual subclasses showed significant intergroup differences. In comparison with the CJN group, the CJA group demonstrated lower relative abundances of LPC, MLCL, SPH, TG, and FA but increased levels of MePC, PC, and PS. Compared to the results in this study, previous findings in BALB/cJ mice revealed opposite trends for LPC, MLCL, and FA following acute *T. gondii* infection [20]. These subclass-level discrepancies may be attributable to differences in the immune responses of the two mouse strains to *T. gondii* infection [33]. Therefore, *T. gondii* infection may exert multifaceted effects on host lipid composition by differentially modulating the metabolic fluxes of various lipid subclasses [34]. Screening and analysis of differentially abundant lipids yielded 136 potential biomarkers associated with *T. gondii* infection. Significantly altered lipids fell into multiple subclasses, such as PE, PC, MePC, LPC, TG, and Cer. Among them, mammalian cell membranes contain PC and PE as their most abundant GPs [35]. Abnormal intracellular PC/PE molar ratios can affect membrane fluidity, signal transduction, and inflammatory responses, thereby linking closely to metabolic disease progression [36]. PE species, such as 18:0 22:6 PE, have been proposed as potential biomarkers of *T. gondii* infection [34]. The significant upregulation of specific PE species in colonic contents during acute infection may therefore reflect not only host membrane shedding but also altered phospholipid remodeling in response to infection. Cer is a core molecule in SP metabolism, as it shapes membrane structure and acts as a second messenger mediating apoptosis, inflammation, and stress responses [37]. Cer accumulation during *T. gondii* infection has been linked to host cell apoptosis and the regulation of intracellular parasite replication [38]. Differential Cer subclass changes in colonic contents suggest that *T. gondii* infection may interfere with SP metabolic pathways. Such interference may affect colonic barrier function and the local immune microenvironment, thereby regulating the infection process.

Among the differentially abundant lipids, the most significantly enriched pathways identified by pathway enrichment analysis were choline metabolism in cancer and GP metabolism. It should be noted that these pathway enrichments represent bioinformatic predictions derived from the differential lipid dataset, rather than experimentally validated pathway activities. Previous studies have associated disturbances in GP and choline metabolism with significant downregulation of glycerophosphocholine phosphodiesterase 1 (GPCPD1) and glycerophosphodiester phosphodiesterase 1 (GDE1). This downregulation leads to elevated total choline levels, a metabolic feature closely linked to disease progression and therapeutic resistance [39]. The differentially abundant lipids involved in the significantly enriched pathways were mainly LPA(16:0) and LPC(18:1). Network analysis identified LPA(16:0) and LPC(18:1) as central nodes connecting multiple metabolic pathways. Thus, they may serve as key hubs in the remodeling of colonic lipid metabolism in response to *T. gondii* infection.

LPA and LPC are key intermediate products of GP metabolism. LPA activates multiple downstream signaling pathways (e.g., MAPK, Akt, and Notch) through binding to its specific G protein-coupled receptors (GPCRs), thereby regulating cell proliferation, migration, survival, and inflammatory responses [40]. LPA also modulates epithelial cell proliferation, survival, and migration while restoring intestinal epithelial barrier function [41]. LPA5 has been identified as a key regulator of intestinal barrier integrity, promoting claudin-4 expression in intestinal epithelial cells via Rac1 and Stat3 activation [42]. Perturbation of LPA signaling, however, promotes inflammation and malignant transformation. LPC(18:1) has been significantly associated with neutrophil infiltration, apoptosis, and oxidative stress markers [43]. The downward trends in both LPA(16:0) and LPC(18:1) detected in this study were possibly related to infection-altered activity of specific lysophospholipases or LPA phosphatases. Decreased serum levels of multiple LPC species, including LPC 16:1, 18:0, and 18:3, have also been observed in patients with active inflammatory bowel disease [44]. This parallel observation between IBD and acute *T. gondii* infection suggests that LPC downregulation may represent a shared metabolic feature of intestinal inflammation rather than a pathogen-specific response. However, this interpretation is based on bioinformatic pathway inference rather than direct enzymatic measurements. The perturbed LPA signaling pathway has been confirmed to promote inflammation and tumorigenesis. These observations raise the possibility that LPA/LPC metabolic disturbances induced by *T. gondii* infection may contribute to infection-associated intestinal pathology [41].

ROC analysis revealed the good predictive ability of PE(20:1e_22:4), PE(16:0_22:6), Hex1Cer(m18:0_20:4), and Hex1Cer(t17:0_22:6) for *T. gondii* infection. PE has been proposed as a potential biomarker and treatment target for *T. gondii* infection [34]. Among the four exploratory candidate markers, PE(20:1e_22:4) and PE(16:0_22:6) showed significant upregulation in the infected group, with an AUC of up to 0.9722. This high diagnostic performance positions these lipids as exploratory candidate markers of infection-associated membrane remodeling and exploratory candidate biomarkers for *T. gondii* infection. However, these promising AUC values (0.9722 and 0.9444) were obtained in a discovery dataset and may be overoptimistic. These lipids should therefore be considered exploratory candidate biomarkers requiring independent validation. Hex1Cer(m18:0_20:4) and Hex1Cer(t17:0_22:6) were also significantly upregulated in the infected group. Hex1Cer is an important SP metabolism intermediate involved in cell recognition, signal transduction, and inflammatory regulation [45]. In a fish model exposed to multi-walled carbon nanotubes, hepatic Hex1Cer, along with other sphingolipids (Cer, SM), was markedly altered, and its dysregulation was associated with oxidative stress and apoptosis [46]. Moreover, Hex1Cer is aberrantly expressed in various infectious diseases and inflammatory bowel diseases, making it an important lipid mediator reflecting intestinal inflammatory status [47]. Lipid metabolic alterations have also been observed in other intestinal parasitic infections. *Cryptosporidium parvum* infection leads to a significant decrease in PC in the intestinal mucosa of calves. Meanwhile, PI(36:1) has been identified as a common lipid biomarker for multiple apicomplexan parasites, suggesting that GP changes may be a shared feature of apicomplexan infections [48]. In contrast to the decreased PC observed in *Cryptosporidium* infection, the present study demonstrated a more extensive elevation of GPs. This difference may reflect that although intestinal parasitic infections commonly disrupt lipid metabolism, the specific lipidomic signatures may be parasite-dependent. These lipids are biologically plausible candidates for infection-associated markers and warrant further investigation in validation cohorts.

A previous study using the ME49 strain in BALB/cJ mice also observed an elevation of glycerophospholipids (GPs) at the lipid class level, which is consistent with our findings. However, notable differences emerged at the network level: that BALB/cJ study identified LPC(20:4) and LPA(18:0) as core hubs, whereas our network analysis identified LPA(16:0) and LPC(18:1) as central nodes [20]. This discrepancy may reflect strain-specific metabolic adaptations. Furthermore, serum-based studies of *T. gondii* infection have primarily reported perturbations in amino acid and tricarboxylic acid (TCA) cycle metabolism [49]. In contrast, the colonic content lipidome in our study was more prominently characterized by changes in GPs and SPs, highlighting the tissue-specific nature of the host metabolic response. Future studies incorporating multiple time points and infection routes would help further elucidate the temporal and spatial dynamics of these lipid alterations.

The lipid alterations identified in this study deepen our understanding of the pathogenesis of *T*. *gondii*. As the parasite relies on host lipids for replication, targeting its lipid metabolic pathways may represent an effective anti-parasitic strategy. Previous studies have shown that disrupting parasite sphingolipid synthesis (e.g., with Aureobasidin A) can block parasite replication without affecting host cells [45]. In addition, modulating the host lipid environment also exhibits anti-parasitic potential. Interfering with cholesterol metabolism (e.g., with AY9944) can inhibit parasite growth [47]. This study observed significant changes in lipid profiles, including elevated glycerophospholipids and reduced sphingolipids. These findings suggest that the affected pathways may serve as potential targets for host-directed immunomodulation or as biomarkers for monitoring therapeutic efficacy. However, the present study is descriptive, and future functional studies are needed to assess whether modulating these lipid changes can effectively limit infection or influence parasite transformation. In summary, our lipidomic data and existing literature support several biologically plausible mechanistic interpretations, including membrane remodeling, altered choline metabolism, and LPA/LPC-mediated inflammation. However, as this was an observational study lacking direct assessment of enzyme activity, inflammatory mediators, or host gene expression, these proposals require future functional validation. The elevated GPs and PE species likely originate primarily from shed host epithelial cells due to infection-induced barrier disruption [12]. In addition, host secretions and parasite-derived lipids (via synthesis or EVs) may also contribute to the altered lipid profile [25]. Distinguishing these sources requires future tissue-specific or parasite-specific tracing studies.

Several limitations inherent in this study must be acknowledged. First, only the acute infection stage was considered, and chronic-phase changes could not be captured. Second, the use of a single mouse strain (C57BL/6J), only female animals, and one parasite strain (ME49) limits the generalizability of our findings. Given the major biological differences among *T. gondii* strains (types I, II, and atypical), it remains unclear whether the observed lipidomic signature reflects a general acute-infection feature or is strain-dependent. Multi-strain comparisons are needed to distinguish universal from strain-specific alterations. Third, the absence of concurrent gut microbiota analysis prevented a full distinction between microbial and host contributions to the detected lipid changes. Fourth, detailed information on infection severity (parasite burden, clinical signs, body weight changes, and histopathological assessment) was not systematically recorded. Consequently, it is difficult to distinguish direct parasite effects from secondary inflammatory or systemic responses. Fifth, the analyzed material consisted of colonic contents rather than colonic tissue or serum; colonic contents are a complex mixture influenced by diet, microbiota, shed epithelium, host secretions, and possibly parasite products. Sixth, the candidate biomarkers were identified and evaluated in the same discovery dataset without targeted validation or independent cohort verification, which may introduce optimistic bias. In addition, the sample size was relatively limited, and potential confounding factors such as food intake, weight loss, and systemic inflammation were not controlled for. Future multi-strain, multi-sex, multi-time-point studies with systematic clinical and histopathological assessment, tissue-specific and targeted lipidomics validation, and independent cohorts are warranted to further validate and extend our findings.

## 5. Conclusions

By leveraging untargeted lipidomics, this study revealed alterations in the lipid profile of colonic contents of C57BL/6J mice following acute *T. gondii* infection. A total of 136 lipids were detected with significant changes following infection, and GP metabolism was identified as a core feature of infection-induced intestinal metabolic disturbance. LPA(16:0) and LPC(18:1) were found as key nodes mediating pathway aberrations. PE(20:1e_22:4), PE(16:0_22:6), Hex1Cer(m18:0_20:4), and Hex1Cer(t17:0_22:6) were identified as exploratory candidate lipid markers for *T. gondii* infection that require independent validation. These findings further the understanding of the intestinal pathogenesis of toxoplasmosis while establishing a theoretical basis for lipid metabolism-oriented host-directed intervention strategies.

## Data Availability

The datasets supporting the findings herein are included within this article. The raw LC-MS/MS data have been deposited in the MetaboLights repository under accession number [MTBLS15624].

## Supplementary Materials

The following supporting information can be downloaded at: https://www.mdpi.com/article/10.3390/microorganisms14092077/s1, Figure S1: Quality control and system stability assessment. Overlaid total ion chromatograms (TIC) of QC samples in positive (A) and negative (B) ion modes; (C) Distribution of coefficients of variation (CV) for all detected lipids; Table S1: Lipid analysis data of colonic contents from C57BL/6J mice with acute *Toxoplasma gondii* infection; Table S2: List of 136 differentially abundant lipids in colonic contents of C57BL/6J mice with acute *Toxoplasma gondii* infection.
